# Bioinformatics searching of diagnostic markers and immune infiltration in polycystic ovary syndrome

**DOI:** 10.3389/fgene.2022.937309

**Published:** 2022-08-31

**Authors:** Xinrui Yao, Xiuxia Wang

**Affiliations:** Center of Reproductive Medicine, Shengjing Hospital of China Medical University, Shenyang, China

**Keywords:** polycystic ovary syndrome, bioinformatics analysis, diagnostic markers, CIBERSORT, immune infiltration, potential therapeutic compounds

## Abstract

Polycystic ovary syndrome (PCOS) is one of the most common endocrine diseases in reproductive-aged women, and it affects numerous women worldwide. This study aimed to identify potential diagnostic markers and explore the infiltration of immune cells in PCOS, contributing to the development of potential therapeutic drugs for this disease. We identified five key genes: CBLN1 (AUC = 0.924), DNAH5 (AUC = 0.867), HMOX1 (AUC = 0.971), SLC26A8 (AUC = 0,933), and LOC100507250 (AUC = 0.848) as diagnostic markers of PCOS. Compared with paired normal group, naïve B cells, gamma delta T cells, resting CD4 memory T cells, and activated CD4 memory T cells were significantly decreased in PCOS while M2 macrophages were significantly increased. Significant correlations were presented between the five key genes and the components of immune infiltrate. The results of CMap suggest that four drugs, ISOX, apicidin, scriptaid, and NSC-94258, have the potential to reverse PCOS. The present study helps provide novel insights for the prevention and treatment of PCOS, and immune cell infiltration plays a role that cannot be ignored in the occurrence and progression of the disease.

## Introduction

Polycystic ovary syndrome (PCOS), defined as one of the most common endocrine abnormalities, is characterized by hyperandrogenism, chronic ovulatory dysfunction and polycystic ovaries and has become a global health burden (Escobar-Morreale, 2018). PCOS is not only a high risk factor for infertility but is also a leading cause of insulin resistance, type 2 diabetes, obesity, hypertension, metabolic syndrome, adverse cardiovascular risk disease, and endometrial cancer (Chan et al., 2017; Cooney and Dokras, 2018). Recently, a diagnosis of PCOS has been mainly based on Rotterdam criteria and clinical symptoms (Ehrmann, 2005); thus, a precise diagnosis of PCOS remains lacking. Because the mechanisms of development and progression of PCOS remain unclear, many patients are misdiagnosed or experience a missed diagnosis (March et al., 2010). Hence, it is necessary to explore biomarkers for the diagnosis of PCOS, so as to improve the prognosis of patients with PCOS by timely detection and intervention.

Recently, a plethora of studies have found that chronic low-grade inflammation has a critical relationship and interaction with PCOS (Barrea et al., 2018; Gong et al., 2018; Li et al., 2019). The discovery of leukocytosis in polycystic ovaries may provide the first clue that polycystic ovaries were a pro-inflammatory condition (Bukulmez and Arici, 2000; Shi et al., 2013). The results of Gong et al. showed that, the expression of IFN-c, a cytokine produced by Th1, was significant increased and Th1/Th2 ratio were significantly higher in PCOS patients than control group (Gong et al., 2018). Regulation of Granulosa cells (GCs) and immune cells is impaired in PCOS patients, may accelerating anovulation (Dewailly et al., 2016). Hence, it is of great value to analyze the infiltration of immune cells and the relationship between infiltrating immune cells and hub genes in order to elucidate the molecular mechanism of PCOS. CIBERSORT is an analytical method used to characterize immune cell composition and expression based on RNA sequencing (RNA-seq) data from samples (Newman et al., 2015). An increasing numbers of studies used CIBERSORT as a computational algorithm for the infiltration of immune cells in various diseases such as pediatric acute myocarditis (Kawada et al., 2021), colorectal cancer (Ye et al., 2019), melanoma (Huang et al., 2020), and lung adenocarcinoma (Mo et al., 2020). However, few studies have used this algorithm to explore the relationship between immune cell infiltration and PCOS.

In our study, clinical information and gene expression of patients with PCOS were downloaded from the Gene Expression Omnibus (GEO) database. Genes differentially expressed between healthy controls and patients with PCOS were screened out by constructing a weighted gene co-expression network analyses (WGCNA) network and using a LASSO method. We then used a CIBERSORT algorithm to analyze immune infiltration between RNA-seq data from patients with PCOS and normal controls. Through the CMap database, we further explored the potential drugs for PCOS treatment, predicting the compounds that can be used to treat PCOS based on the differentially expressed genes (DEGs) in PCOS.

## Materials and methods

### Data collection and processing

GSE34526 (Kaur et al., 2012) and GSE137684 datasets were downloaded from the GEO database. The GSE34526 dataset contained mRNA expression profiles from the GCs of seven PCOS samples and three healthy controls, while the GSE137684 dataset contained mRNA expression profiles from the GCs of eight PCOS samples and four healthy controls. We used a “*limma*” package (Ritchie et al., 2015) for R to search out DEGs. We set significance expressed as a *p* value <0.05 and |log2FC|>1.

### Consensus weighted gene co-expression network analysis and LASSO algorithm for searching the target markers

We detected co-expressed gene modules and explored the association between gene networks and phenotypes of interest through WGCNA analysis (Langfelder and Horvath, 2008). WGCNA analysis involved four major steps: 1) Constructing co-expressed gene networks and gene modules from gene expression datasets (GSE34526) and (GSE137684). Genes with a variance in the top 5000 were selected for co-expression network analysis and further analyzed. The soft threshold power, R-squared, was set to 3; 2) We transformed the acquired weighted adjacency matrix into a topological overlap matrix to assess network connectivity. 3) Topological overlap matrix was then used to perform an average-linkage hierarchal clustering method generating a clustering tree. Diverse cluster tree branches represented different gene modules, which were labeled with different colors. 4) Based on the weighted correlation coefficient of genes, we classified all genes by their expression patterns. Genes with similar patterns were classified into a module; in this way, all genes were divided into dozens of modules. We used the receiver operating characteristic (ROC) curves to assess diagnostic value of genes in PCOS. The “*glmnet*” package was used to apply the LASSO algorithm to reduce the variables.

### Functional analysis of mRNAs

The R package “*clusterProfiler*” (Yu et al., 2012) was used to conduct functional annotations and explore the functional relevance of DEGs. We used Gene Ontology (GO) and KEGG (Kyoto Encyclopedia of Genes and Genomes) to assess the relevant functional categories through a “*clusterProfiler*” package. Combined *p* < 0.05 and *q* < 0.05 were considered significant enrichment.

### Evaluation of immune cell infiltration

Based on the RNA-seq data of different subgroups, CIBERSORT algorithm was used to estimate the abundances of 22 leukocyte subtypes. The interactive relationship between immune cells was analyzed by using a “*corrplot*” package to further investigate the influence of interaction between immune cells. A “*vioplot*” package was used to draw a violin plot to visualize immune cell distribution. We used Spearman’s rank correlation coefficient for the correlation analysis of gene expression and the content of immune cells, identifying a *p* value <0.05 as statistically significant.

### Statistical analysis

All statistical analyses were performed using R software (version 4.0). The differences between different groups were compared by a Mann–Whitney–Wilcoxon test. *p* Value <0.05 was considered statistically significant.

## Results

### Preprocessing of data and identification of DEGs

**FIGURE 1:**
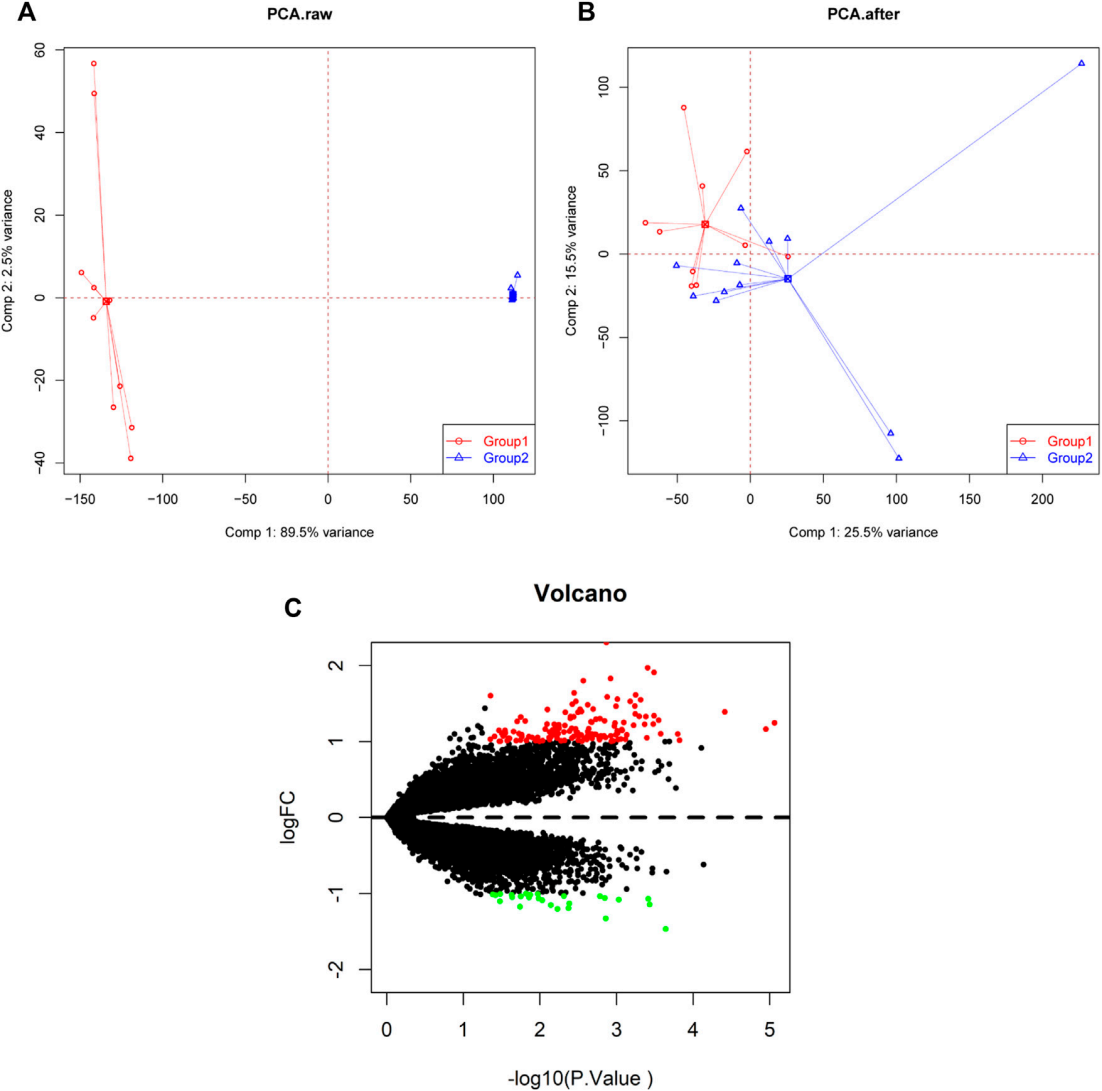
PCA cluster plot before and after batch effect adjustment and volcano plot of DEGs. **(A)** PCA cluster plot of the GSE34526 and GSE137684 datasets without batch effect adjustment. **(B)** PCA cluster plot of the GSE34526 and GSE137684 datasets with batch effect adjustment. **(C)** Volcano plot of DEGs between PCOS and normal samples; red shows upregulated DEGs, black shows no significant DEGs, and green shows downregulated DEGs. PCA, principal component analysis; DEGs, differentially expressed genes; PCOS, Polycystic ovary syndrome.

GSE34526 and GSE137684 datasets were downloaded from the GEO database to explore the key genes that may have important roles in PCOS. All uploaded data passed the integrity check. Characteristics of the two datasets included in the analysis was shown in Supplementary Table S1. A spatial variant apodization algorithm was used to remove batch effects among different datasets. We normalized and processed the merged gene expression matrix, and then showed it in a principal component analysis (PCA) plot before and after normalization (Figures 1A,B). Using “*limma*” package for R to filter out DEGs, 168 DEGs between response and non-response PCOS groups were obtained according to the criteria of adjusted |log2FC|>1 and *p* value <0.05. The 142 upregulated genes and 26 downregulated genes are shown in a volcano plot and heat map (Figure 1C, Supplementary Figure S1).

### Functional enrichment analyses of DEGs

**FIGURE 2:**
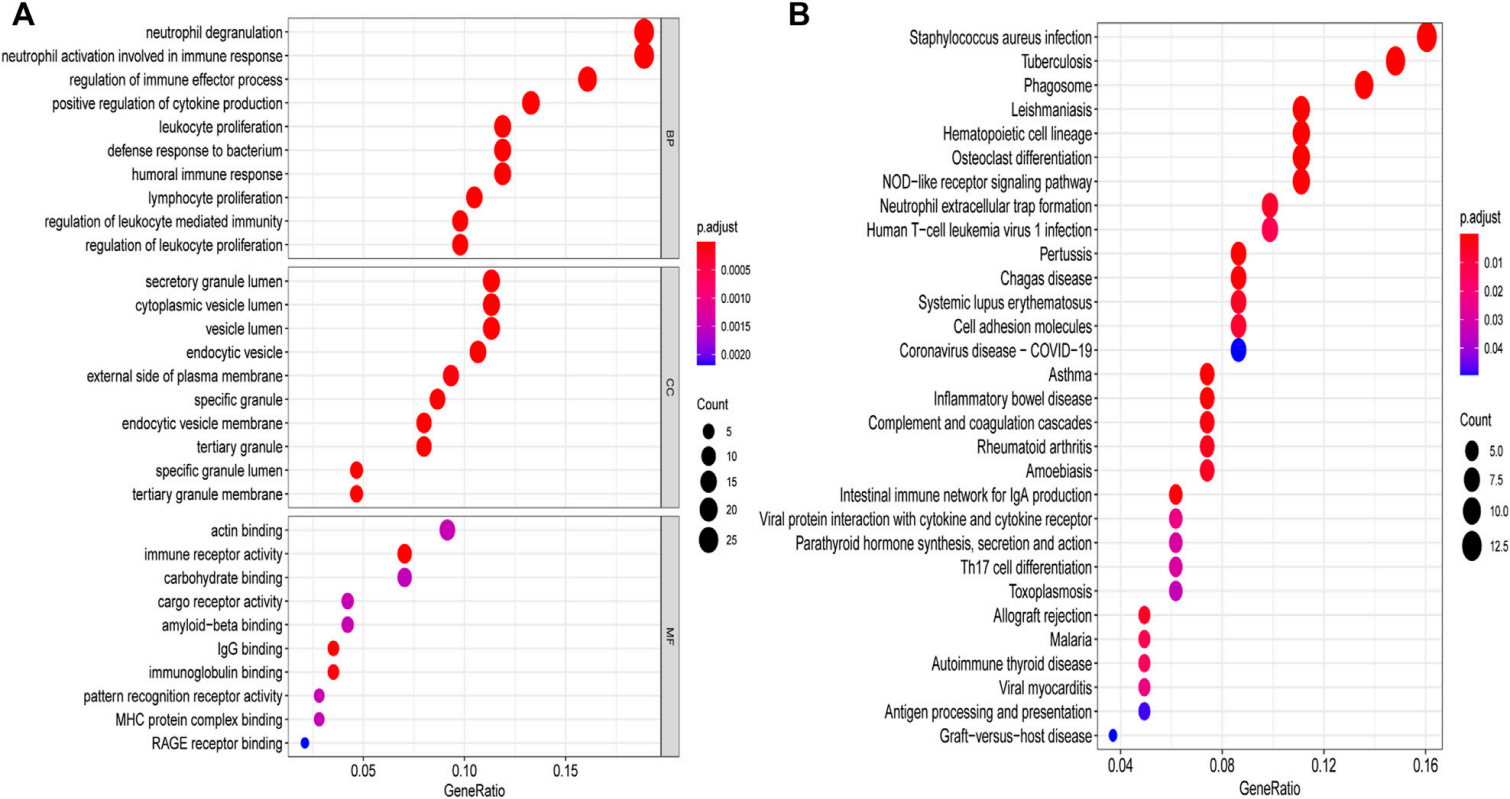
GO and KEGG analyses of DEGs. **(A)** GO enrichment analysis, bubble plot of the functional enrichment analysis including BP, CC, and MF. **(B)** KEGG enrichment analysis, bubble plot of the functional enrichment analysis. GO, Gene Ontology; KEGG, Kyoto Encyclopedia of Genes and Genomes; DEGs, differentially expressed genes; BP, biological processes; CC, cellular component; MF, molecular functions.

We performed pathway analysis on the 168 differential genes, of which pathways were involved in PCOS development, as presented in Figure 2. Results from GO analysis showed that DEGs were mainly related to specific biological processes “neutrophil degranulation”, “neutrophil activation involved in immune response”, “regulation of immune effector process”, “positive regulation of cytokine production”, “humoral immune response”, “lymphocyte proliferation” “regulation of leukocyte mediated immunity”, and “regulation of leukocyte proliferation”. In aspect of cellular component, DEGs were highly involved in “secretory granule lumen”, “cytoplasmic vesicle lumen”, “vesicle lumen”, “endocytic vesicle”, “external side of plasma membrane”, “specific granule”, “endocytic vesicle membrane”, “tertiary granule”, “specific granule lumen” and “tertiary granule membrane”. Immune receptor activity, IgG binding and immunoglobulin binding were the major molecular functions of these DEGs. The results of KEGG analysis showed that DEGs were mainly correlated to pathways such as “*Staphylococcus* *aureus* infection”, “phagosome”, “Neutrophil extracellular trap formation”, “Systemic lupus erythematosus”, “Inflammatory bowel disease”, “Complement and coagulation cascades”, “Rheumatoid arthritis”, and “Intestinal immune network for IgA production”.

### Weighted gene co-expression network analyses combined with LASSO analyses of DEGs in patients with and without PCOS

**FIGURE 3:**
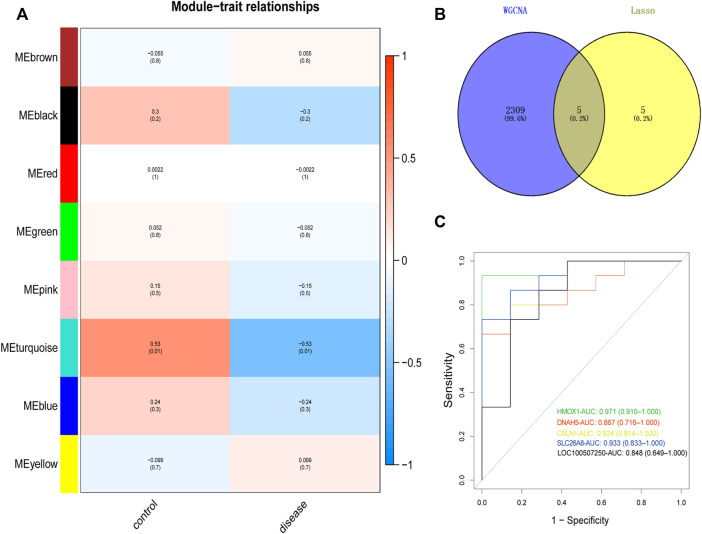
Identification and ROC curves of diagnostic markers. **(A)** Heat map showed positive and negative correlation of the gene module in the PCOS and normal samples, respectively. **(B)** Venn plot was performed to show the intersection of potential target genes using WGCNA and Lasso. **(C)** The ROC curve of the diagnostic power of five target genes (*CBLN1*, *DNAH5*, *HMOX1*, *SLC26A8*, *LOC100507250*). ROC curves, receiver operating characteristic curves; PCOS, Polycystic ovary syndrome; WGCNA, weighted gene co-expression network analyses; LASSO, least absolute shrinkage and selection operator.

We constructed co-expression networks through weighted gene co-expression network analyses (WGCNA) based on GSE34526 and GSE137684 datasets to explore associated co-expression networks in PCOS. A total of 5,000 differentially expressed genes in eight gene co-expression modules, such as black module (244 DEGs), blue module (696 DEGs), brown module (566 DEGs), green module (364 DEGs), pink module (152 DEGs), red module (284 DEGs), turquoise module (2,314 DEGs), and yellow module (380 DEGs) were screened. The threshold power of β was determined by the function “sft$powerEstimate” (Supplementary Figure S2). Through a further estimate of the relationship between modules and characteristics, we identified that the turquoise module had the closest correlation with PCOS (cor = 0.53, *p* = 0.01; Figure 3A). The genes of the turquoise module with the highest correlation were intersected with 10 gene pairs screened by LASSO (Supplementary Figure S3), in the end, we identified five important target genes (*CBLN1*, *DNAH5*, *HMOX1*, *SLC26A8*, *LOC100507250*; Figure 3B).

### Screening and verifying candidate biomarkers by ROC curves

The receiver operating characteristic (ROC) curve is a graphical plot described by plotting the true versus false positive probabilities as a function of the discrimination threshold (Cao and López-De-Ullibarri, 2019). The area under the curve (AUC) has been proposed as a summarized accuracy index (Martínez-Camblor et al., 2021). Hence, we used ROC curve analysis to predict candidate biomarkers; AUC values indicated a good predictive performance. Of all these hub genes, *CBLN1* (AUC = 0.924), *DNAH5* (AUC = 0.867), *HMOX1* (AUC = 0.971), *SLC26A8* (AUC = 0.933), and *LOC100507250* (AUC = 0.848) were highly predictive of the occurrence and development of PCOS (Figure 3C).

### Immune cell infiltration analysis

**FIGURE 4:**
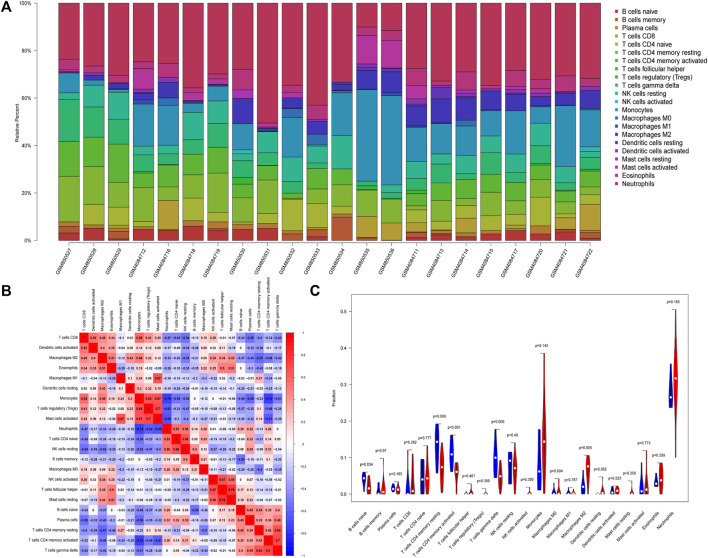
Differential distributions of immune cells in PCOS and normal samples. **(A)** Bar plot of fractions of 22 immune cells. **(B)** Heat map of correlations between 22 types of infiltrated immune cells in PCOS and normal samples. **(C)** Violin plot depicted the different infiltrated immune cells between PCOS and normal samples.

We analyzed the relationship between DEGs and immune cells in datasets related to PCOS. The potential molecular mechanism of DEGs influencing the progression of PCOS was further explored. To ensure the accuracy of the analysis, we excluded samples with a calculated *p* value >0.05. The results were illustrated in a bar plot (Figure 4A), different immune cells were colored differently, and the sum of the immune scores for each sample was equal to one. The proportions of 22 infiltrating immune cells were weakly-to-strongly correlated in PCOS. Mast cells resting and T cells follicular helper showed the strongest positive correlation (Pearson correlation = 0.76), while Neutrophils and Monocytes showed the strongest negative correlation (Pearson correlation = 0.74); Macrophages M2 indicated moderate negative correlation with resting CD4 memory T cells (Pearson correlation = 0.57) while gamma delta T cells showed moderate positive correlation with activated CD4 memory T cells (Pearson correlation = 0.70) and resting CD4 memory T cells (Pearson correlation = 0.64) (Figure 4B).

**FIGURE 5:**
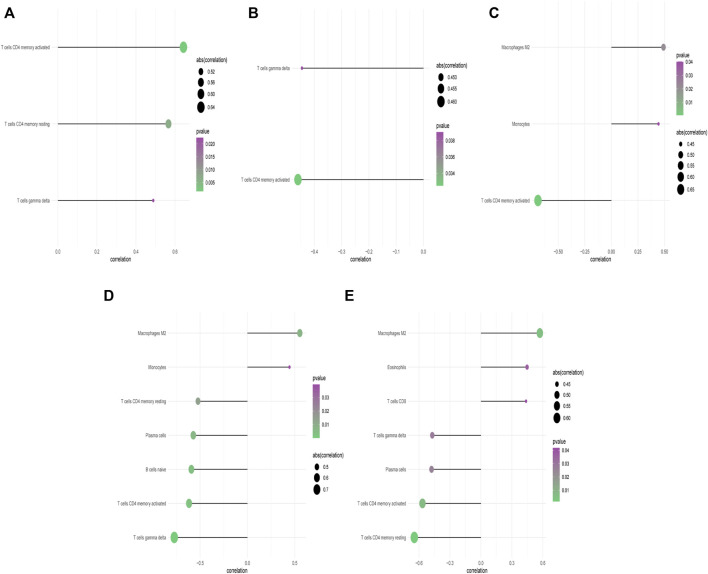
Correlation between *CBLN1*, *DNAH5*, *HMOX1*, *SLC26A8*, *LOC100507250* and infiltrating immune cells. **(A)** Correlation between *CBLN1* and infiltrating immune cells. **(B)** Correlation between *LOC100507250* and infiltrating immune cells. **(C)** Correlation between *DNAH5* and infiltrating immune cells. **(D)** Correlation between *HMOX1* and infiltrating immune cells. **(E)** Correlation between *SLC26A8* and infiltrating immune cells.

Our study showed that compared with paired normal group, naïve B cells, gamma delta T cells, resting CD4 memory T cells, and activated CD4 memory T cells were significantly decreased in PCOS while M2 macrophages were significantly increased, which met with expected results (Figure 4C). The *CBLN1* was correlated positively with activated CD4 memory T cells, resting CD4 memory T cells and gamma delta T cells (Figure 5A). *LOC100507250* was negatively correlated with gamma delta T cells and activated CD4 memory T cells (Figure 5B). The *DNAH5* showed a significant positive correlation with M2 macrophages and monocytes but was negatively related to activated CD4 memory T cells (Figure 5C). The *HMOX1* showed a significant positive correlation with M2 macrophages and monocytes while was negatively related to resting CD4 memory T cells, plasma cells, naïve B cells, and activated CD4 memory T cells and gamma delta T cells (Figure 5D). The *SLC26A8* was positively related to M2 macrophages, Eosinophils and CD8 T cells but was negatively correlated to gamma delta T cells, Plasma cells, activated CD4 memory T cells and resting CD4 memory T cells (Figure 5E).

### Identification of potential drugs for PCOS

**FIGURE 6:**
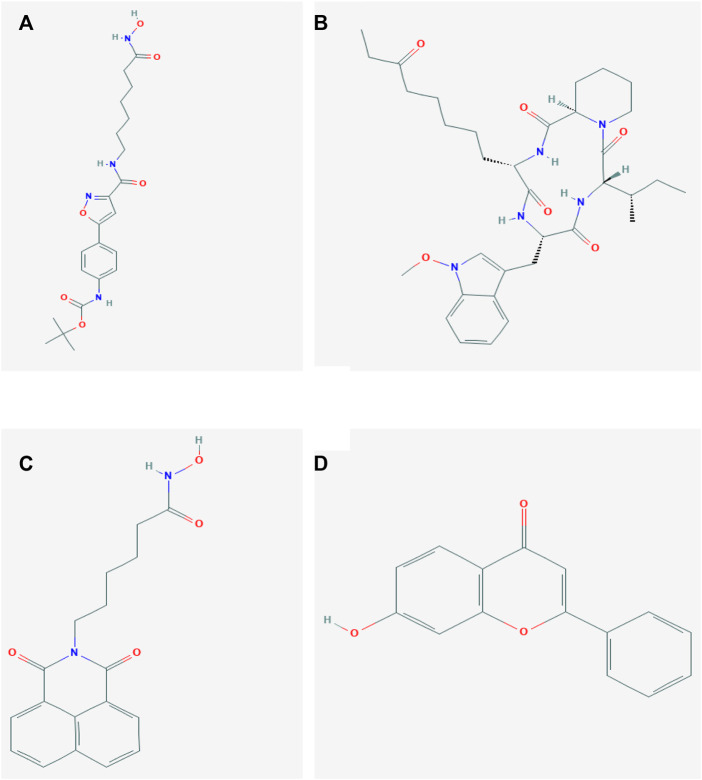
Chemical structures of the four molecules. **(A)** Structure diagram of ISOX. **(B)** Structure diagram of apicidin. **(C) **Structure diagram of scriptaid. **(D)** Structure diagram of NSC-94258.

We further explored potential drugs for PCOS treatment through a connectivity map (CMap) (https://clue.io). CMap is a computational screening approach which predict biochemical interactions of small molecules with their respective targets (Lamb et al., 2006). Genes with significant differential expression in PCOS were analyzed to predict compounds that might have a therapeutic effect in PCOS. It was found that ISOX, apicidin, scriptaid, and NSC-94258 might have a potential effect in reversing the development of PCOS. In addition, we used a PubChem database (http://pubchem.ncbi.nlm.nih.gov/) to explore the structure diagram of four compounds shown in Figure 6.

## Discussions

Polycystic ovary syndrome is a heterogeneous and complicated endocrinopathy that has both adverse reproductive and metabolic implications for women of reproductive age (Dumesic et al., 2015). Unfortunately, existing epidemiologic and/or basic research data have not been sufficient in providing the foundation needed to derive an evidence-based definition of the syndrome (Lizneva et al., 2016). Because of the insufficiency of diagnostic indicators, patients with PCOS often miss a suitable time for diagnosis and treatment. Recent studies have showed that immune infiltration is critically correlated with PCOS (Barrea et al., 2018; Gong et al., 2018; Li et al., 2019). Hence, it is of great significance to identify specific diagnostic markers and analyze immune cell components in PCOS. In the current study, we attempted to identify diagnostic markers for PCOS and provide the description of the role of immune cell infiltration in PCOS through the CIBERSORT. In addition, we performed CMap analysis to investigate potential therapeutic agents for PCOS.

According to our results, five DMGs (*CBLN1*, *DNAH5*, *HMOX1*, *SLC26A8*, *LOC100507250*) were the potential diagnostic markers of PCOS. PCOS patients who seeking the help of assisted reproduction technology because of ovulation dysfunction often have poor quality and the low maturation rate of oocytes *in vitro* (Qiao and Feng, 2011). The expression of *CBLN1* is thought to increase with follicle development and reach a peak before ovulation (Zhang et al., 2018); therefore, we suggest that the *CBLN1* expression level in GCs might affect the quality of oocytes. According to the literature, it has been shown that the mutations in dynein axonemal heavy chain 5 (*DNAH5*) have been accepted as one of the most common causes of Primary ciliary dyskinesia (PCD) (Failly et al., 2009). The complex PCD phenotype involving various organ systems is explained by dysfunction of motile cilia and flagella (Hornef et al., 2006). Hyperandrogenism is one of the most common complications of PCOS (Escobar-Morreale, 2018). As the results showed by Jackson-Bey et al. (2020), human fallopian tube epithelium (hFTE) exposed to the 2 nM testosterone displayed slower cilia beating, inhibited estrogen signaling. We speculate that in the high levels of androgen environment of PCOS patients, *DNAH5* may mutate which leading to reduced cilia motility. Increased oxidative stress is a key mechanism of obesity-related insulin resistance (Furukawa et al., 2004), playing an important part in PCOS patients with obesity (Cortón et al., 2007; Cortón et al., 2008). *HMOX1* is a facultative gene induced by inflammatory mediators and oxidative stress. It is suggested that the upregulation of *HMOX1* might have anti-inflammatory effects and protect cells from oxidative damage (Mannerås-Holm et al., 2014). Hence, it is speculated that the increased expression of *HMOX1* in PCOS might represent a compensatory or protective mechanism for reducing oxidation and inflammation. *SLC26A8* mainly affects sperm motility and capacitation events by regulating Ca^2+^, Cl^−^, and HCO^3−^ influxes in the testes (El Khouri and Touré, 2014), which are homologous with ovaries (Houmard et al., 2009). Ca^2+^, Cl^−^, and HCO^3−^ influxes are responsible for increasing the intracellular cAMP concentration and the subsequent activation of PKA and phosphorylation cascades (Xie et al., 2006). Here is our inference: *SLC26A8* may be downregulated in PCOS, affecting Ca^2+^, Cl^−^ influxes and HCO^3−^ activation thus leading to the development of cystic or multifollicular ovaries (Johannesson et al., 1998; Chen et al., 2012). Through analysis of the human tissue-specific expression by genome-wide integration of transcriptomics and antibody-based proteomics, *LOC100507250* is highly expressed in ovaries and lymph nodes (Fagerberg et al., 2014). This indicated that *LOC100507250* linked the ovary function and immune system. Combined with our results, although research on *LOC100507250* is very limited, it is our speculation that *LOC100507250* may interact with immune lymphocytes, especially gamma delta T cells and activated CD4 memory T cells, involving in the pathological process of polycystic ovary.

The CIBERSORT algorithm showed that an increased infiltration of M2 macrophages and a decreased infiltration of naïve B cells, gamma delta T cells, resting CD4 memory T cells, and activated CD4 memory T cells might be related to the occurrence and progression of PCOS. Insulin resistance is one of the most common characteristics of PCOS. Chronic exposure to insulin caused mouse macrophages to develop insulin resistance characterized by increased glycolysis and a unique M2-like phenotype (Ieronymaki et al., 2019), which explains changes in macrophage response and a trained immune status associated with PCOS. Gamma delta T cells, a primary source of the pro-inflammatory cytokine, interleukin (IL)-17a, are generally resident in adipose tissue (Zúñiga et al., 2010). Interleukin-17 has an inhibitory effect on adipogenesis, and regulates the accumulation of adipose tissue (AT) and glucose metabolism in mice (Zúñiga et al., 2010). In contrast to our study, the greater numbers and proportions of peripheral naïve B cells were found to exist in PCOS than in the control group (Xiao et al., 2019). We speculate that the transient reduction of naive B cells was caused by accelerated differentiation into memory cells after activation. Previous studies have shown reduced staining of the T lymphocyte markers, CD3, CD4, and CD45RA, in the ovarian endometrium of PCOS (Wu et al., 2007). The expression of CD4^+^ T cells in PCOS was significantly decreased compared with that of a normal ovulation group (Li et al., 2019). One of the possible explanations is that a high expression of programmed cell death protein 1 in CD4^+^ T cells in the follicular fluid of PCOS may not induce the activation or recruitment of T cells, leading to the failure of dominant follicle selection and development (Benedict et al., 2008; Chikuma, 2016). Programmed cell death protein 1 is an inducible receptor that can inhibit the antiviral T cell response by interacting with two ligands, programmed death ligand 1 (PD-L1) and PD-L23. T cells may reasonably assist the survival of follicles by providing trophic growth factors or by inhibiting adverse immune activity (Wu et al., 2007). The above function requires sufficient and appropriately distributed T lymphocytes. If T cell populations are deficient or lacking, this might lead to an abnormality in follicle selection and development, which may promote the occurrence of PCOS. Interleukin-2 is involved in the development of CD4^+^ T cell memory and studies have shown that patients with PCOS had lower IL-2 (Krishna et al., 2015; Demir et al., 2019). Interleukin-2 produced by adjacent CD4^+^ T cell populations triggered CD62L expression, which served as a marker for T cell memory (Tubo et al., 2013), indicating that IL-2 mediates local signals between adjacent CD4^+^ T cells, which can affect T cell memory fates. The above literature evidence combined with our analysis shown that these immune cells play a significant role in PCOS.

By analyzing the correlation between DMGs and immune cells, we found that *DNAH5* showed a positive correlation with M2 macrophages and a negative correlation with activated CD4 memory T cells; *HMOX1* was positively correlated with M2 macrophages while it negatively correlated with resting CD4 memory T cells, naïve B cells, activated CD4 memory T cells, and gamma delta T cells; *SLC26A8* was positively correlated with M2 macrophages, and negatively correlated with gamma delta T cells, activated CD4 memory T cells and resting CD4 memory T cells; *CBLN1* showed a positive correlation with activated CD4 memory T cells, resting CD4 memory T cells CD4 and gamma delta T cells; *LOC100507250* showed a negative correlation with gamma delta T cells and activated CD4 memory T cells. Kabil Kucur et al. (2016) reported that PCOS with hyperandrogenism is associated with prolonged nasal mucociliary clearance time (NMCT), which may lead to respiratory tract and middle ear infections. The middle ear infections is characterized by the upgraded macrophages cells and dendritic cells according to an rat study (Jecker et al., 1996). We found that *DNAH5* showed a positive correlation with M2 macrophages. Combined with the above results, the mutation of *DNAH5* in PCOS may lead to the PCD with infections, which characterized by the upgraded macrophages M2 cells. As we mentioned above, *HMOX1* was correlated with oxidative stress and might had anti-inflammatory effects in obese PCOS patients, which was consistent with our results: *HMOX1* showed a negative relation with resting CD4 memory T cells, naïve B cells, activated CD4 memory T cells, and gamma delta T cells. CBLN1 contains a globular C1q domain characteristic of the C1q family of target recognition proteins of the classical complement pathway (Yuzaki, 2008), and the deletion of C1q impairs CD4 T cell immunity (Kerdidani et al., 2022). Forouhi et al. (2016) provided evidence that C1q/tumornecrosis factor (TNF)-related protein 9 levels were higher in PCOS patients as compared to their age and BMI-matched controls, indicating that C1q may be an intermediate mediator of the interaction between *CBLN1* and immune cells in PCOS. Although there were currently no related studies on *SLC26A8* and immune infiltration in PCOS, *SLC26A8* was correlated to many diseases (Dirami et al., 2013; El Khouri and Touré, 2014), being involved in some regulatory pathways, including the immune response, T cell activation, Toll-like receptor binding, granulocyte activation, and GTPase regulator activity (Han et al., 2021). *LOC100507250* is highly expressed in ovaries and lymph nodes, hence we assumed that *LOC100507250* may interact with immune lymphocytes, especially gamma delta T cells and activated CD4 memory T cells, involving in the pathological process of PCOS. These speculations require further research to clarify the complex relationships between *SLC26A8*, *LOC100507250*, and immune cells.

We predicted that ISOX, apicidin, scriptaid, and NSC-94258 would be useful in the treatment of PCOS. ISOX and apicidin are histone deacetylase 6 (HDAC6) inhibitors and scriptaid is a potent HDAC8 inhibitor (Janaki Ramaiah et al., 2017). Previous studies showed that HDAC6 and HDAC8 promoted insulin resistance in animal models (Winkler et al., 2012; Tian et al., 2015). Insulin resistance is a very common complication in PCOS patients, according to the WHO criteria for defining insulin resistance, about 75% of PCOS women have impaired insulin sensitivity (Tosi et al., 2017). The insulin resistance have the capacity to induce both the endocrine and reproductive traits of PCOS (Moghetti and Tosi, 2021). We inferred that ISOX, apicidin, and scriptaid may play a preventive role in the PCOS by improving insulin resistance. In addition, scriptaid may alleviate PCOS by inhibiting the secretion of TC androstenedione (Vanhaecke et al., 2004). NSC-94258, as an antineoplastic agent, its protein targets are *AKR1B1*, *CYP19A1*, *HSD17B1*. Aldo-ketoreductase family 1, member B1 (AKR1B1), can prevent complications of diabetes and improve insulin sensitivity by catalyzing the reduction of glucose to sorbitol (Zhan et al., 2019; Syaifie et al., 2022). In GCs, androstenedione and testosterone act as substrates and are converted by *CYP19A1* to estrogen, the mutation of *CYP19A1* may lead to the occurrence and the development of hyperandrogenemia in PCOS (Kumariya et al., 2021). *HSD17B1*, a gene encode enzymes which are critical to ovarian steroidogenesis, showed a decreased expression in PCOS compared with the control group (Lerner et al., 2019). These results indicated that NSC-94258 may ameliorate the reduced insulin sensitivity and hyperandrogenemia of PCOS by targeting and regulating *AKR1B1*, *CYP19A1*, *HSD17B1*. We assumed that the correlation between DMGs and potential drugs stems from insulin resistance. It was reported that variants within or near *DNAH5* modified glucose response in acute coronary syndromes (Ellis et al., 2015). *HMOX1* expression negatively correlated with insulin resistance as assessed by the homeostasis model assessment of insulin resistance (HOMA-IR) (Shakeri-Manesch et al., 2009). Through the rat experiment, Strowski et al. (2009) identified CER, a neuromodulatory hexadecapeptide that originates from *CBLN1*, as an insulinostatic factor. These assumptions need further research to explore the complex relationship between DMGs and potential drugs.

It must be admitted that our research has certain limitations. Our results are based on public databases and computational algorithms, which involves the second mining and analysis of previously released datasets. Second, several diagnostic markers, immune cells, and potential drugs are more strongly associated with insulin resistance. Although insulin resistance is an important characteristic of PCOS, further experiments are needed to characterize the role of diagnostic markers and immune cells in this disease. Although the results of prior research are in accord with our findings, these still need to be verified in future experiments.

In a nutshell, we found that *CBLN1*, *DNAH5*, *HMOX1*, *SLC26A8*, and *LOC100507250* are diagnostic markers of PCOS. Our analysis, based on a devolution algorithm, showed significant differences in the cellular composition of infiltrating immune cells in PCOS. In particular, M2 macrophages, naïve B cells, gamma delta T cells, resting CD4 memory T cells CD4, and activated CD4 memory T cells might be related to the occurrence and progression of PCOS. Further research is needed on these DEGs and immune cells may provide a feasible direction for the diagnosis and immunotherapy of PCOS in the clinic.

## Data Availability

Publicly available datasets were analyzed in this study. This data can be found here: GSE34526, GSE137684.

## Supplementary material

The Supplementary Material for this article can be found online at: https://www.frontiersin.org/articles/10.3389/fgene.2022.937309/full#supplementary-material

Supplementary Figure S1 Heat map of the 168 DEGs in PCOS vs normal patients. The vertical axis represents the 168 regulated genes, and the horizontal represents PCOS and normal samples.

Supplementary Figure S2 Determine soft-threshold power for WGCNA.: Left: Analysis of the scale-free fit index for various soft-threshold powers β. Right: Analysis of the mean connectivity for various soft-threshold powers.

Supplementary Figure S3 LASSO regression method for searching the DEGs. The plot determined the Coefficient of LASSO regression model.
