# Supplementary figures and images for: Bioinformatics searching of diagnostic markers and immune infiltration in polycystic ovary syndrome
Source: Front Genet. 2022 Aug 31;13:937309. doi: 10.3389/fgene.2022.937309 (PMC9471256; doi:10.3389/fgene.2022.937309)

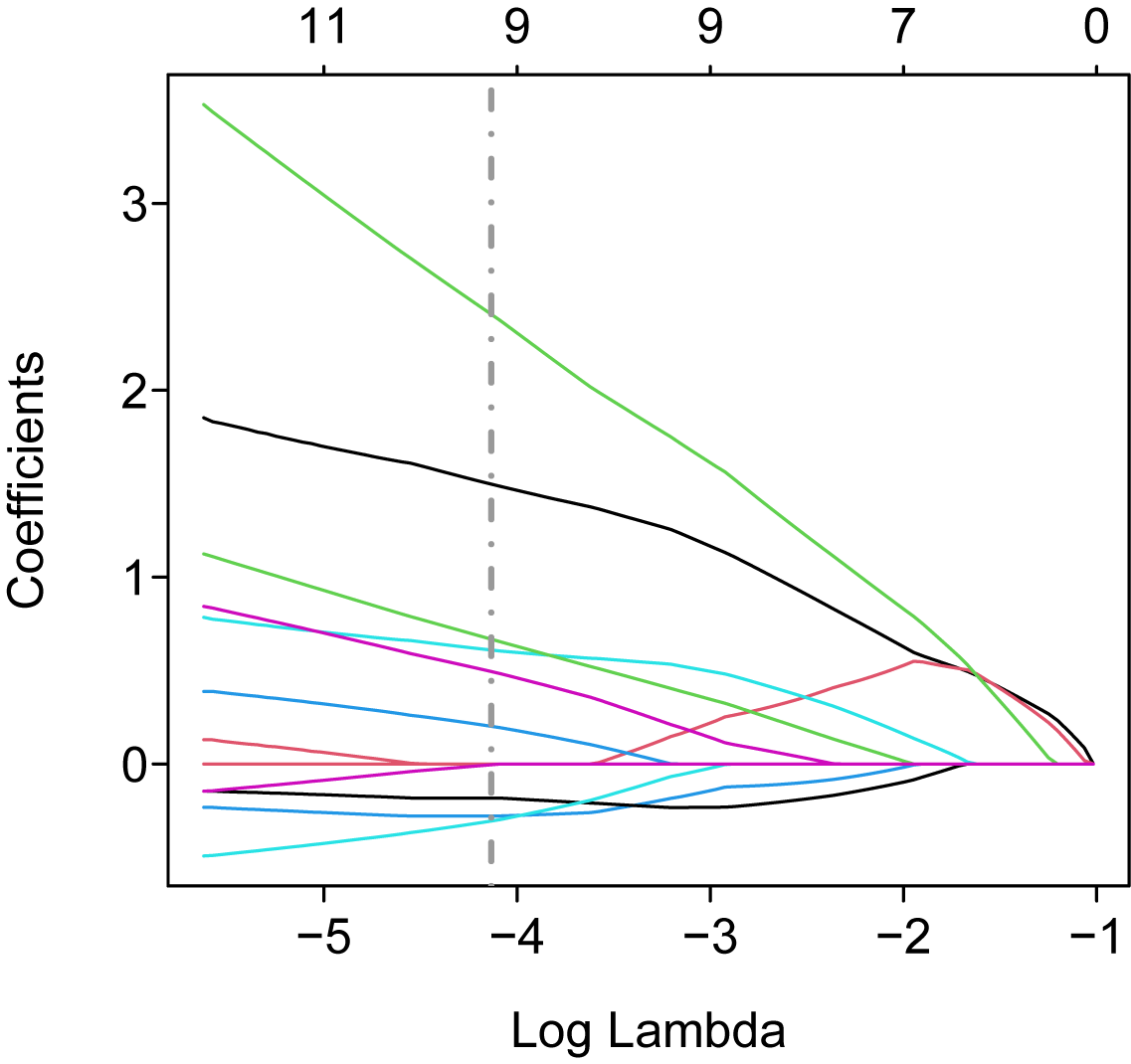

Supplement: Supplementary file 3 [file Image3.TIF]

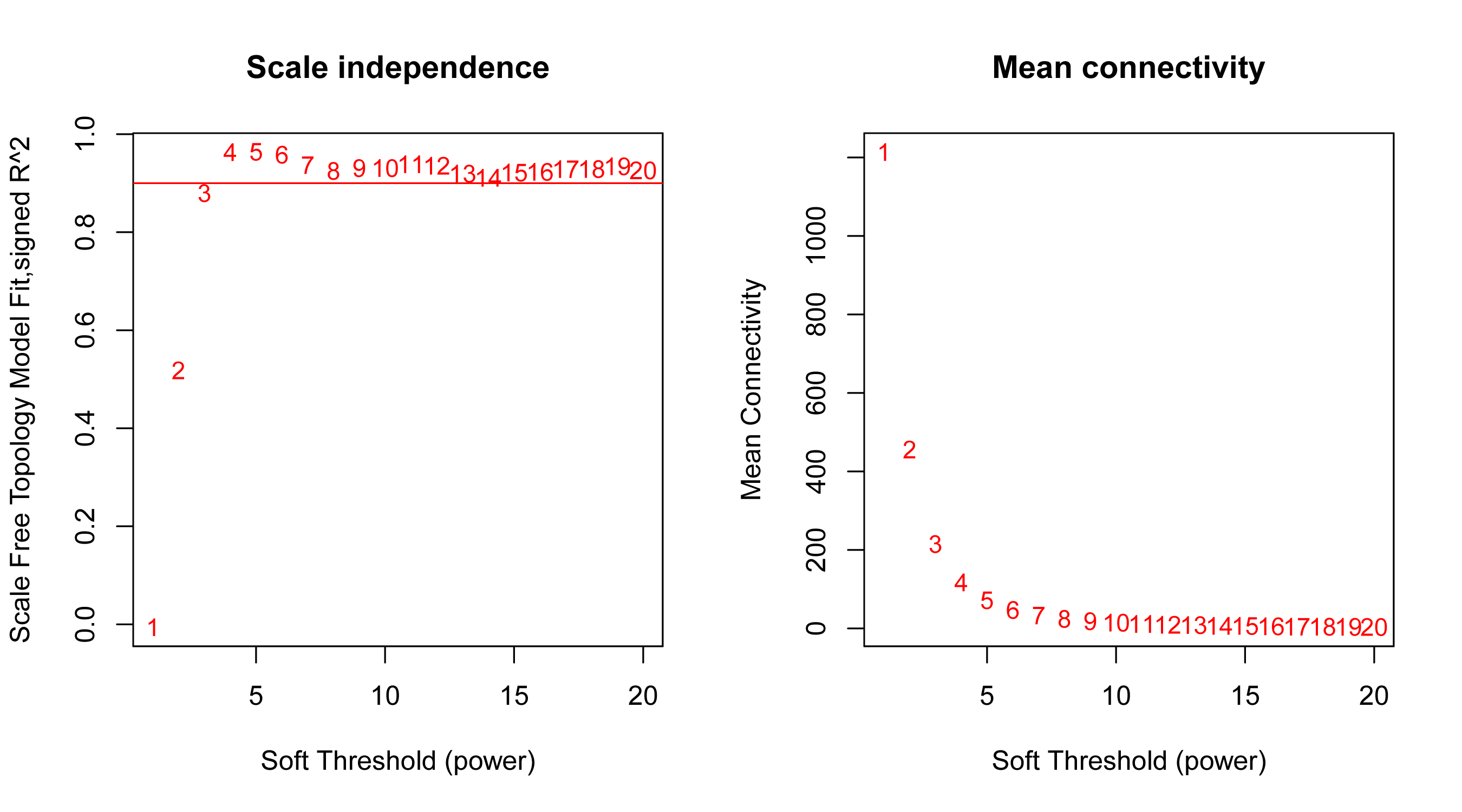

Supplement: Supplementary file 4 [file Image2.TIF]

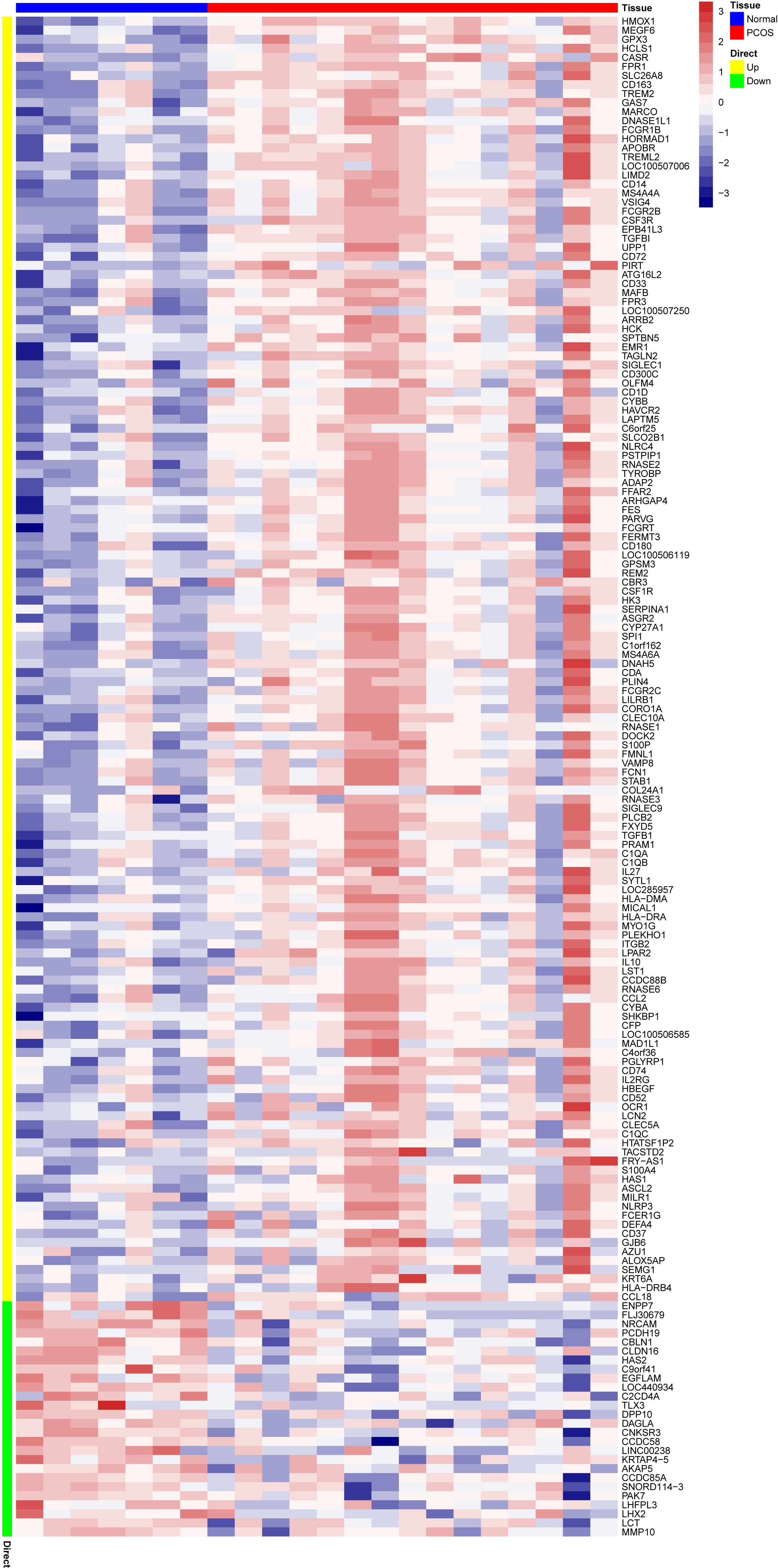

Supplement: Supplementary file 6 [file Image1.TIF]
