# Supplementary material for: Bioinformatics searching of diagnostic markers and immune infiltration in polycystic ovary syndrome
Source: Front Genet. 2022 Aug 31;13:937309. doi: 10.3389/fgene.2022.937309 (PMC9471256; doi:10.3389/fgene.2022.937309)
Supplement: Supplementary file 7 [file Table5.DOCX]

**Supplementary Table 1:** Characteristics of the two datasets included in the analysis.

| GEO dataset | GSE34526 | GSE137684 |
| --- | --- | --- |
| Normal, n | 3 | 4 |
| PCOS, n | 7 | 8 |
| Tissue type (s) | Granulosa cell | Granulosa cell |
| Country | India | China |
| PMID | 22904171(Kaur et al., 2012) | Missing |
| Platforms | GPL570 | GPL17077 |
| Study design | Human granulosa cells were isolated from ovarian aspirates from normal and PCOS women undergoing IVF and for each sample, RNA was extracted and hybridized to an Affymetrix GeneChip. | A total of 18 subjects were enrolled and 12 of the subjects were divided into three groups (control, NA PCOS and HA PCOS) with 4 subjects in each group. Recruitment took place from December 2015 to January 2016. |

PCOS, Polycystic Ovary Syndrome.

KAUR, S., ARCHER, K. J., DEVI, M. G., KRIPLANI, A., STRAUSS, J. F., 3RD & SINGH, R. 2012. Differential gene expression in granulosa cells from polycystic ovary syndrome patients with and without insulin resistance: identification of susceptibility gene sets through network analysis. *J Clin Endocrinol Metab,* 97**,** E2016-21.
